# Sex-related differences in death control of somatic cells

**DOI:** 10.1111/jcmm.12047

**Published:** 2013-03-20

**Authors:** Claudia Giampietri, Simonetta Petrungaro, Antonio Filippini, Elio Ziparo

**Affiliations:** a Department of Anatomy, Histology,Forensic Medicine and Orthopedics-Section of Histology and Medical Embryology, Istituto Pasteur-Fondazione Cenci Bolognetti, Sapienza University of RomeRome, Italy

*Dear Editor*:

In 2001, The United States Institute of Medicine (IOM) Committee on Understanding the Biology of Sex and Gender Differences concluded that ‘Sex…should be considered when designing and analysing studies in all areas and at all levels of biomedical and health-related research…’ and stated an apparent paradox *i.e*.: ‘every cell has a sex’ 1. Sex is defined as ‘the classification of living things, generally as male or female according to their reproductive organs and functions assigned by chromosomal complement’ whereas gender is defined as ‘a person's self representation as male or female, or how that person is responded to by social institutions based on the individual's gender presentation. Gender is rooted in biology and shaped by environment and experience’ 1.

It is unchallenged that there are health differences between males and females and that social and cultural factors could contribute to the observed differences. Anyway, the sex-dependent differences also have a biological base which sometimes has not been deeply investigated. Scientists studying health differences between male and female aim to both considering social/cultural environment and investigating biological/molecular mechanisms different between sexes. Some experimental studies have elucidated important differences in cell death control 2. A sex disparity, in fact, has been shown both in the propensity to apoptosis and in the activation of the autophagic pathway. In the context of cell fate control, hormones represent important regulators of both apoptosis and autophagy. In the cardiovascular system, for example, oestrogens inhibit cardiomyocyte apoptosis by decreasing reactive oxygen species production and increasing intracellular antioxidants 3. Oestrogens may also indirectly control autophagy as they up-regulate urocortin 4, a neuropeptide hormone able to inhibiting autophagy in cardiomyocytes. Conversely, increasing evidence suggests possible adverse effects of androgens on the vasculature showing that androgens, as opposed to oestrogens, may worsen vascular dysfunction in men, thus contributing to sex-based differences in cardiovascular diseases 5.

However, it is currently emerging that some cell death programs are differentially controlled by sex-related hormone-independent cellular genetics. Differences in cell death sensitivity in male and female may then occur in the absence of an hormonal context. This is not an immediately obvious finding; Penaloza C *et al*., 6 have shown that the apoptosis amount differs between the sexes in isolated embryonic cells exposed to similar conditions and this happens at embryonal stages where there are no hormonal influences. Previous studies had reported a sexual dimorphism in embryonic neuronal signal transduction pathways and consequently differences in cell survival 7. Death pathways in XX and XY cells have been poorly investigated as most studies have been performed on established cells lines often irrespective of their male or female origin. Recently, using freshly isolated cells from male and female individuals gave important information on sex disparity in cell fate control. Such sex specificity has been in part clarified thanks to cell culture models where sex steroids can be removed from the media. Even sex-related differences in caspase activation have been found to be independent on hormone exposure. More in detail, cell death occurring in cortical neurons after ischaemia proceeds predominantly *via* an apoptosis-inducing factor-dependent pathway (a caspase-independent pathway) in male neurons while proceeds *via* a cytochrome C-dependent pathway (a process mediated by caspase activation) in female neurons 8. In this context, a sex-specific microRNA expression after ischaemia has been described in *in vivo* studies. In particular, it has been demonstrated that microRNA-23a, by binding the mRNA of the caspase inhibitor named XIAP, induces its translational repression in females, leading to enhanced caspase signalling in the ischaemic female brain. This effect has been shown to be independent of circulating oestrogen levels 9. Sex differences in ischaemic brain injury and cerebrovascular regulation have been observed in clinical and experimental studies and an important determinant of such differences is also represented by the integrity of endothelial cells. In fact, endothelial function is improved in women compared with men**,** contributing to female cellular higher resistance after ischaemic brain injury. Gupta NC *et al*. 10 showed that female cerebrovascular endothelial cells express lower level of soluble epoxide hydrolase and consequently have higher levels of vasoprotective epoxyeicosatrienoic acids as compared with male endothelial cells. This study therefore presents a novel additional mechanism underlying differences between male and female cells in apoptotic response after oxygen-glucose deprivation, contributing to explain higher resistance observed in females as compared with males. This study remarks again that differences between male and female cells do not necessarily depend on the hormonal context but may be inherent the cells. We believe that this apparently paradoxical concept has not been sufficiently highlighted in the scientific literature. The present ‘Letter to the Editor’ therefore aims at underlining such an important issue which deserves more attention and discussion in the researchers' community. A practical consequence of sex-dependent discrepancies in cell death control is that cellular response to any stimulus or treatment, in any physiological or pathological context, may well depend on the sex of the cell line used; journals guidelines should therefore require authors to state in any case the sex of the cell lines used in any *in vitro* study. In addition, at least to some extent, sex-matched or sex-unmatched cell controls may be necessary in many experimental settings. In conclusion, sex-related differences in cell death mechanism may have strong implications for experimental studies and sexual dimorphism dependent on chromosomal rather than hormonal differences have important implications for planning preclinical studies and clinical interventions.
